# Optimal Fertilizer Application Reduced Nitrogen Leaching and Maintained High Yield in Wheat-Maize Cropping System in North China

**DOI:** 10.3390/plants11151963

**Published:** 2022-07-28

**Authors:** Xiaosheng Luo, Changlin Kou, Qian Wang

**Affiliations:** 1 Henan Key Laboratory of Agricultural Eco-Environment, Institute of Plant Nutrition, Resources and Environmental Sciences, Henan Academy of Agricultural Sciences, Zhengzhou 450002, China; koucl@126.com; 2 Key Laboratory for Agricultural Environment of Ministry of Agriculture and Rural Affairs, Institute of Environment and Sustainable Development in Agriculture, Chinese Academy of Agricultural Sciences, Beijing 100081, China; wangqian02@caas.cn

**Keywords:** N leaching, in situ leakage pond, interannual variation, grain yield, soil nitrate N, North China Plain

## Abstract

Agricultural nitrogen (N) non-point source pollution in the North China Plain is a major factor that affects water quality and human health. The characteristics of N leaching under different N application conditions should be further quantified accurately in winter wheat (*Triticum aestivum* L.) and summer maize (*Zea mays* L.) rotation farmland in North China, and a basis for reducing the risk and evaluation of N leaching in this area. A three-year field experiment was conducted using an in situ leakage pond method at a typical farmland in Henan in 2017–2020. Crop yield, soil nitrate N residues, and N utilization were also studied during the study period. Five N fertilizer rates were established with 0 (CK), 285 (LN), 465 (MN), 510 (MNO), and 645 (HN) kg N ha^−1^ for one rotation cycle. MNO was applied with chemical and organic fertilizers. The concentration of nitrate N in the soil leaching solution of CK, LN, MN, MNO, and HN was 0.81-, 1.49-, 3.65-, 5.55-, and 7.57-fold that of the World Health Organization’s standard for underground drinking water. The exponential relationship between the N application rate and leaching was obtained when the annual N input exceeded 300 kg ha^−1^, and the N leaching rate increased greatly. The leaching rate of nitrate N in the total N was 50.6–82.4% under different treatments of N application. The combination of chemical and organic fertilizers treatment (MNO) reduced the amount of N that was leached in dry years. The nitrate leaching amount of summer maize accounts for 83.0%, 49.4%, and 72.0% of the total nitrate leaching amount of the whole rotation cycles in 2017–2020. LN and MN were recommended as the optimized N application here (285–465 kg N ha^−1^) with the two-season rotation grain yield of 17.2 ton ha^−1^ (16.5–17.9 ton ha^−1^) and nitrate N leaching of 21.6 kg ha^−1^ (12.6–30.5 kg ha^−1^).

## 1. Introduction

Nitrogen (N) fertilizer produced via the Haber–Bosch process reached 96 Tg worldwide in 2010, accounting for the largest portion of anthropogenic reactive N. N fertilizers are highly important to feed the rapidly growing world population, but their heavy application introduces some environmental hazards, such as nitrate leaching [1,2,3]. Nitrate leaching is one of the main pathways through which N fertilizer is lost in the farmland. The loss of nitrate N in crop systems by leaching can comprise >10% of the total input of N fertilizer [4,5]. Nitrate leaching from agriculture is a major source of concern because it can lead to ground and surface water contamination, which may affect human health and aquatic wildlife [6]. Developed countries have encountered these problems and conducted relevant research to introduce effective countermeasures to control N leaching from agriculture [3,7]. The European Union adopted the Nitrates Directive in 1991 that aimed to reduce and prevent the water pollution caused by nitrate from agricultural sources. The use of organic fertilizers is restricted to 170 kg N ha^−1^ in zones that are vulnerable to nitrate with a regulated time window for its application. In addition, the supply of mineral fertilizer is restricted from exceeding the crop requirements [8,9]. The use of catch crops and organic agriculture has been recommended and can help maintain high water quality [10,11,12].

Food production in China has developed dramatically over the past 40 years. The production of N fertilizer contributed to the increase in grain yield, but the rate of increase of N fertilizer application far exceeded the rate of increase in grain yield. The production of N fertilizer in China comprises approximately one-third of the production throughout the world; as a result, serious agricultural non-point source pollution has been a substantial concern for the whole society [13,14,15]. The North China Plain is the most important region for grain production in China, which supplies 40% and 60% of the national maize and winter wheat production, respectively [16]. High yield and good economic benefits are obtained by farmers by using large amounts of N fertilizer, resulting in a high amount of nitrate N leaching in north China [17,18,19]. A groundwater survey in Henan, Shandong, Beijing, and Tianjin in North China showed that the nitrate N content in groundwater was high, and 14.2–34.1% of the samples exceeded the standard rate of drinking water of 11.3 mg N L^−1^, which was established by the World Health Organization (WHO) [20,21]. Quantification of N leaching and its relationship with crop yield and N use efficiency is an important method to achieve a balance between low N leaching risk and high crop yields [22,23]. Some studies have been conducted on N leaching in different areas of North China, including the use of a Lysimeter shelter facility, and model simulation, among others. However, the researchers primarily focused on single crops, such as wheat or maize, that lacked systematic long-term observations and had large uncertainties in their quantitative methods [24,25,26]. Furthermore, although nitrate N leaching is a major part of the leaching losses of N in the field, less attention has been paid to total N leaching in this area.

Henan is located southwest of the North China Plain and is an important grain-producing region in China, providing a quarter of the country’s wheat output and one-tenth of that of corn. The consumption of chemical N fertilizer in Henan reached 1900 kt in 2019 and was the highest among all the provinces [27]. However, the amount and coefficient of N leaching loss in this region remain unclear, and no related evaluation indices have been established.

In the present study, an in situ leakage pond method was used to research the characteristics of N leachate under different types of N fertilization management in farmland that rotated winter wheat and summer maize. The soil nitrate N residue, grain yield, and N utilization rate were also studied during the experimental period. This study aimed to obtain the characteristics of N leaching in winter wheat and summer maize rotation fields in North China and to establish relevant indicators to assess the risk of N leaching. In addition, this study sought to provide a theoretical basis for realizing high yields of wheat and maize, highly efficient N fertilization, and reduced risk of losing N by leaching in this region.

## 2. Material and Methods

### 2.1. Study Site Description

This study was conducted using ongoing field experiments at the Experimental Station of Henan Academy of Agricultural Sciences, Yuanyang County (35.1° N, 113.7° E), Xinxiang, China. Henan Province is located southwest of the North China Plain. The crops that are grown in the experimental area are primarily a rotation of winter wheat and summer corn. The soil is classified as a saturated eusol (sandy fluvo-aquic soil). The basic soil properties in the experimental site were soil pH of 8.9, organic matter of 10.9 g kg^−1^, total N of 0.63 g kg^−1^, total P of 0.75 g kg^−1^, nitrate N of 18.9 mg kg^−1^, and available potassium of 144.1 mg kg^−1^. The area is affected by a warm temperate continental monsoon climate, the annual rainfall is approximately 600 mm and concentrated in June, July, and August, and the average temperature is 14.0 °C at the experimental site. The annual rainfall fluctuated more strongly during the three-year period of this experiment, with rainfall measurements of 554.4, 346.8, and 572.3 mm. In comparison with the other years, less rainfall was observed in the summer of 2019 (Figure S1).

### 2.2. Experimental Design

The experiment started in 2017 after the harvest of maize (*Zea mays* L.). A rotation system of summer maize and winter wheat (*Triticum aestivum* L.) was adopted in 2017–2020. The three rotation seasons were 2017–2018, 2018–2019, and 2019–2020 (designated RS1, RS2, and RS3, respectively). The chosen cultivars were Zheng 369 for wheat and Dedan 5 for maize. The fertilization treatments that used different rates of N were as follows: zero N rate (CK); low N rate (LN), in which the input of N was assumed to be less than that of the optimal N application; medium N rate (MN), assumed optimal N rate; combined inorganic and organic fertilization (MNO), additional organic fertilizer added to MN; and high N rate (HN), which is a common rate of N that results in a serious excess of N fertilizer.

The amounts of fertilizers applied are provided in Table 1. Three replicates were employed in each treatment, and 15 experimental plots were randomly grouped. Each plot had an area of 40 m^2^. The N fertilizer that was applied was divided into base fertilizer and top dressing. The period of application of the base fertilizer for winter wheat and summer maize was the seeding and seedling stages, respectively, and the top dressing was added at the jointing stage. The ratio of basal N to top dressing N was 4:6. P and K fertilizer were applied once as basal fertilization in the form of monoammonium phosphate and potassium chloride. In addition to P and K fertilizer, commercial organic fertilizer was applied once only in the wheat season at a rate of 2250 kg ha^−1^ (MNO treatment), and the N content was 2%. Both winter wheat and summer maize were irrigated once in 2017/18. In 2018/19, both wheat and maize were irrigated twice. In 2019/20, the winter wheat was irrigated twice, whereas the summer corn was not irrigated. The winter wheat was irrigated with 90 mm. However, the wheat was irrigated a second time with 60 mm in 2019/20. The summer corn was irrigated with 60 mm. Specific irrigation results from 2018 to 2020 are shown in Figure S1. Flooding was used for irrigation.

### 2.3. Sample Collection and Analysis

**Figure 1:**
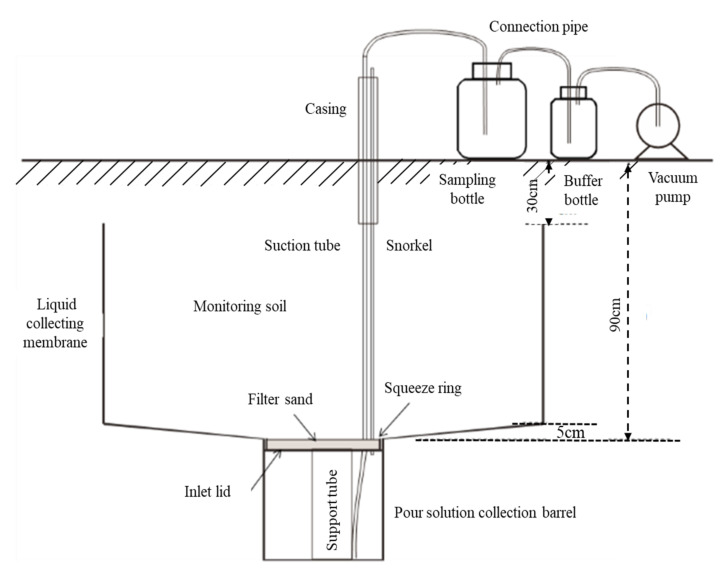
Structural diagram of the field seepage pool to collect the soil leachate.

One in situ field leakage pond was built in each plot of replicates with length, width, and depth of 1.5 m × 0.8 m × 0.9 m. A leaching barrel was installed beneath the pond for leachate collection. The infrastructure was constructed in the fall of 2014. Detailed information on the methods of structure and measurement is described in Figure 1. We excavated a soil profile with a length of 1.5 m, width of 0.8 m, and height of 0.9 m. The excavated soil was stacked on plastic film marked with the soil layer number based on the depth of layers (0–20, 20–40, 40–60, and 60–90 cm) to allow its backfilling in layers. The walls of the soil profile needed to be kept distinct to avoid their collapse during excavation. The bottom of the soil profile was then restructured into an inverted trapezoid that was approximately 3 cm higher than the center to allow the collection of leaching solution in the middle. A small cylindrical section that was 35 cm deep with a diameter of 40 cm was dug in the center of the section to place the eluvial collecting barrel. Intercepting barrels covered with nylon net were installed. The liquid membrane was tailored to set, resulting in a set liquid film around a closed area that was consistent with the size of holes of the box body. It opened at the bottom of the small mouth with a smoke fluid tube that used the liquid film pressure to set on the squeeze film in the leaching solution barrel cover. The cover was cut to cover the last liquid membrane and was spread again on the quartz sand and barrel level. The excavated soil was backfilled in layers in reverse order and compacted while backfilling. The liquid collecting film was closely connected with the four walls of the frame. The backfill process had a small amount of irrigation, resulting in heavy soil. When the backfilling was 30 cm away from the surface, the liquid collecting film was cut off along the surface of the backfilling soil. The ventilation and liquid extraction pipes were placed vertically on the soil surface through the casing. Finally, the uppermost soil was backfilled (Figure 1). After irrigation and heavy rainfall, the leachate solution was collected. If any liquid was collected, the leachate volume was recorded. Samples were stored at 4 °C and subsequently analyzed for nitrate N and total nitrogen (TN). Ten leachate sampling dates were recorded, including 25 March 2018, 27 July 2018, 29 August 2018, 28 August 2018, 28 March 2019, 24 May 2019, 27 July 2019, 07 April 2020, 21 July 2020, and 27 August 2020. One leachate collection was carried out for both RS1 and RS3 and twice for RS2 during the wheat season. The leachate was collected three times, once and twice in RS1-3 during the maize season. The concentration of nitrate N was measured using a continuous-flow analyzer (AutoAnalyzer 3 BRAN+LUEBBE, Norderstedt, Germany), and the concentration of TN was determined by digestion with alkaline potassium persulfate via UV spectrophotometry (UV-2802H, Shanghai, China).

The yield of grain and concentration of N were determined for each plot after harvest. The soil nitrate N was measured by collecting samples of a 2-m layer in the intervals of 20 cm in RS2, extracting the sample with 1 mol L^−1^ KCl, and conducting a flow analysis after shock filtration. The soil pH was determined to be 2.5:1 using the suspension potential method. A semi-trace Kelvin method was used to determine the soil total N. The soil organic matter was determined using the potassium dichromate–external heating method. Total phosphorus in the soil was determined by UV-Vis spectrophotometry. The soil available potassium was determined by flame photometry.

### 2.4. N Leaching and N Utilization Rate Calculation

The N leaching rate was calculated as follows:

(1) $$F=\sum_{i=1}^{n}\frac{Vi*Ci}{S}*f$$

where *F* is the N leaching rate (kg ha^−1^), *n* is the number of leaching times, Vi is the leaching water volume at leaching time i (L), Ci is the leachate concentration of nitrate N at leaching time i, *S* is the monitoring area (1.2 m^2^), and *f* is the transformation ratio from the monitoring area to per hectare.

The N fertilizer partial factor productivity (PFP) and N recovery efficiency (RE) were calculated as follows [28]:

PFP (kg/kg)= Y_f_/R_f_ (2)

where PFP is the N fertilizer partial factor productivity, *Y_f_* is the yield of wheat or maize (kg), and *R_f_* is the N fertilizer application rate (kg).

RE (%)= (TU_N_ − TU_CK_)/TN (3)

where TU_N_ is the total wheat/maize N uptake (kg N ha^−1^) in the treatments with N fertilization, TU_CK_ is the total wheat/maize uptake of N (kg N ha^−1^) in CK, and TN is the total N fertilizer rate (kg N ha^−1^).

N leaching coefficient = N leaching loss/N application rate × 100%(4)

### 2.5. Statistical Analysis

Differences in the data from different treatments of the N leaching rate, crop yield, and N use efficiency were calculated using analysis of variance (ANOVA) by using SPSS 13.0 (SPSS, Inc., Chicago, IL, USA). Significant differences were established at *p* < 0.05. The figures were generated using SigmaPlot 10.0 (Systat, San Jose, CA, USA).

## 3. Results

### 3.1. Concentrations of Leachate Nitrate N and Variation in the TN during Study Period 

**Figure 2:**
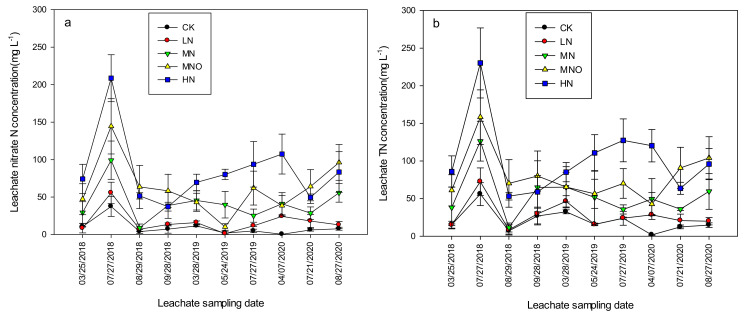
The concentrations of nitrate N and TN in the leached solution were collected during the study period. (**a**) Nitrate N concentrations; (**b**) TN concentrations. TN, total nitrogen. Note: CK, 0 N rate; LN, N input that was assumed to be less than the optimal application of N; MN, assumed to be the optimal N rate; MNO, additional organic fertilizer added to the rates of MN. HN, excess of N fertilizer.

The leachate was collected 10 times during the three-rotation season period, including four times in RS1 (2017/18), three times in RS2 (2018/19), and three times in RS3 (2019/20, Figure 2). The concentration of nitrate N in the CK soil leaching solution was low. One exception was that the concentration of the second leaching solution was 37.4 mg L^−1^, and the concentration of the nine other leaching solutions was only 0.3–11.5 mg L^−1^. The LN of the concentration of nitrate N in the soil leaching solution was close to the CK, and the concentration during the monitoring period was 1.18–55.5 mg L^−1^. The concentrations of nitrate N in the MN, MNO, and HN soil leachate were 8.98–99.2, 10.0–144.4, and 37.2–208.7 mg L^−1^, respectively, in 10 soil leachates collected during the three-year monitoring period. The three-rotation average concentrations of nitrate N were 9.1, 16.8, 41.2, 62.7, and 85.5 mg L^−1^ in the CK, LN, MN, MNO, and HN, respectively. With the increase in N application rate, the nitrate N concentration of the LN, MN, MNO, and HN soil leachates increased by 0.8-, 3.5-, 5.9-, and 8.4-fold compared with CK, respectively. The concentrations of TN leachate showed a similar trend as the concentration of nitrate N with values of 20.6, 28.0, 53.8, 79.9, and 103.2 mg L^−1^. The concentrations of nitrate N and TN in the soil leachate were the highest in 2017/18 and the lowest in 2018/19.

### 3.2. Annual Mean Leaching Loss and Leaching Coefficient of Nitrate N and TN 

**Figure 3:**
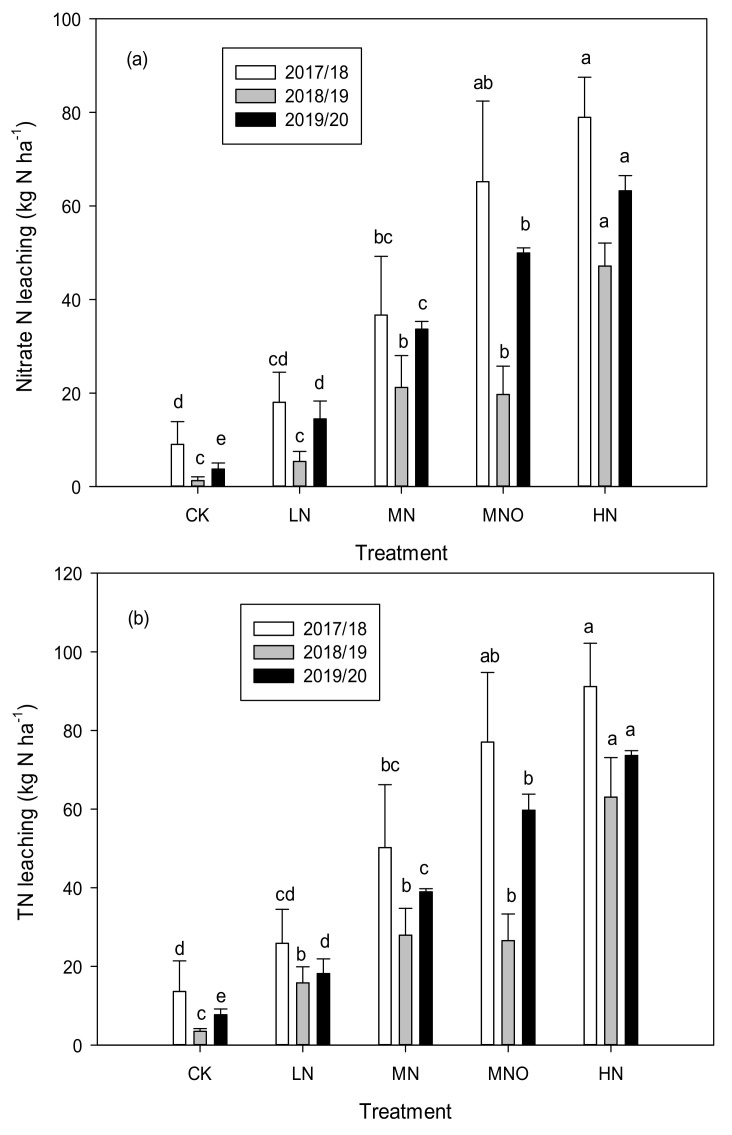
Nitrate N and TN leaching amounts during the three-year rotation of winter wheat and summer maize. (**a**) Nitrate N leaching amount; (**b**) TN leaching amounts. Significant difference was determined among different treatments in the same year. Different lowercase letters indicate significant differences (*p* < 0.05). N, nitrogen; TN, total nitrogen. Note: CK, 0 N rate; LN, N input that was assumed to be less than the optimal application of N; MN, assumed to be the optimal N rate; MNO, additional organic fertilizer added to the rates of MN. HN, excess of N fertilizer.

As shown in Figure 3a, the rate of N fertilization had significant effects on nitrate N leaching. The leaching of nitrate N increased in parallel with the rate of N. The nitrate N leaching amount of HN was the highest, and it was significantly higher than those under CK, LN, and MN treatments in RS1-3. In RS1 and RS2, no significant differences were observed between the CK and LN, and this finding was similar to that of MN and MNO. In comparison with MN, MNO had a higher rate of leaching nitrate N in RS1 and RS3, whereas the rate in RS2 was slightly lower, suggesting annual effects from rainfall. In RS3, significant differences were observed in nitrate leaching among the five N treatments. The leaching of the TN in different N treatments showed the same pattern as those of the nitrate (Figure 3b).

In terms of the leaching of N by TN, the three-year average values were 8.3, 20.0, 39.0, 54.4, and 75.9 kg ha^−1^ in the CK, LN, MN, MNO, and HN, while the values of nitrate N leaching were 4.7, 12.6, 30.5, 45.0, and 63.1 kg ha^−1^, respectively. Nitrate N dominated the TN with average nitrate N-to-TN ratios of 50.6%, 61.1%, 78.5%, 80.8%, and 82.4% under the five treatments.

The N leaching coefficient varied substantially between years. For TN, the leaching coefficient ranged from 9.1% to 15.1%, 5.2% to 9.8%, and 6.4% to 11.7% in different treatments in 2018–2020. For nitrate, the three-year average leaching coefficients of the LN, MN, MNO, and HN treatments were 4.4%, 6.6%, 8.8%, and 9.8%, respectively. During the experiment, the average CK, LN, MN, MNO, and HN concentrations were 1.3, 3.7, 9.2, 13.2, and 18.8 kg N ha^−1^ per nitrate N leaching, and the TN values were 3.4, 7.9, 13.7, 17.6, and 20.4 kg N ha^−1^, respectively.

### 3.3. Nitrate N Residue in a 2 m Soil Layer during the Sampling Period

**Figure 4:**
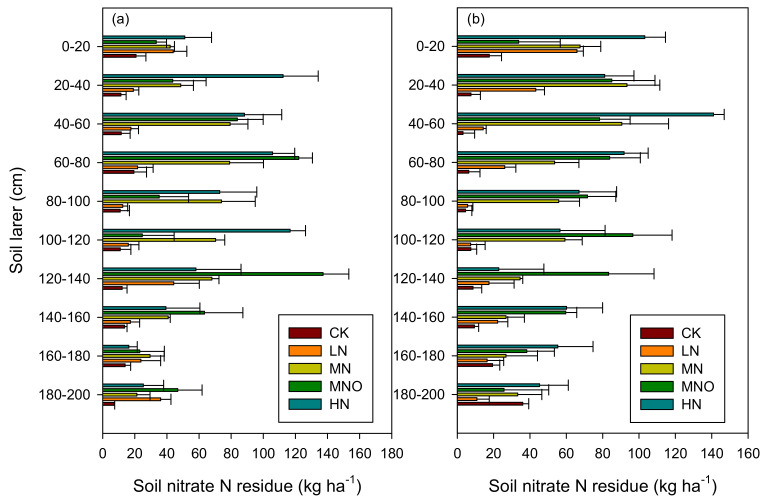
Soil nitrate N content measured at the different soil layers to a depth of 2 m at the time of harvest in rotation season 2 (RS2). (**a**) Wheat season; (**b**) maize season. Note: CK, 0 N rate; LN, N input that was assumed to be less than the optimal application of N; MN, assumed to be the optimal N rate; MNO, additional organic fertilizer added to the rates of MN. HN, excess of N fertilizer.

The nitrate N residue at the 2-m soil layer in five treatments at harvest time in RS2 is shown in Figure 4. Typically, the soil nitrate residue increased in parallel by using N fertilization at both sampling times. The HN treatment had the highest nitrate N residue in the 0–100 cm soil layer, followed by the MNO treatment. The average total nitrate N residues in the 2 m soil layer were 131.8, 252.0, 552.7, 613.1, and 686.0 kg ha^−1^ for the wheat season and 120.1, 229.3, 541.4, 660.0, and 724.1 kg ha^−1^ for the maize season in CK, LN, MN, MNO, and HN, respectively. The range of changes in the LN treatment in different soil layer contents of nitrate N was smaller than those in the other fertilization treatments. The difference of nitrate N residue in the 140–200 cm soil layer under different fertilization treatments was lower than that of the 20–140 cm soil layer under different fertilization treatments.

### 3.4. Winter Wheat and Summer Maize Yield 

In RS1, the wheat yield of MNO was the highest, followed by MN and HN, while LN was the lowest (Table 2). The yield of maize was the highest in MN maize. The yields in MNO and HN reached 10,470.0 and 9,791.1 kg ha^−1^, respectively. In RS2, the yields produced by the five fertilization treatments during the wheat season were in the order of HN > MN > LN > MNO > CK. The order of maize yield was MN > HN > MNO > LN> CK. In RS3, the gaps in yield of wheat and maize were small, except for CK. The LN treatment produced high yields during this rotation year. Overall, no significant difference was observed in yield between MN and MNO and HN. MN produced the highest yields in the maize season. HN did not consistently produce the maximum yield during six crop seasons.

### 3.5. Crop N Uptake and N Utilization Rate of Winter Wheat and Summer Maize 

In general, the N uptake of wheat and maize in RS3 increased in parallel with the N application rate (Table 3). The N uptake of HN for wheat was significantly higher than those of the four other N rate treatments, and the CK was significantly lower than those of the other four. HN, MN, and LN had increased N uptake, but no significant difference was observed among them. LN and CK had a low N uptake. For RE, LN had a significantly higher RE than the other rate of N in the wheat season, whereas no significant difference was observed between HN and MNO. MNO had the highest RE during the maize season, followed by MN. The effects of the N rate on the PFP were similar in both the wheat and maize seasons, and LN had the highest PFP.

## 4. Discussion

### 4.1. N Leaching Characteristics for Winter Wheat and Summer Maize Rotation Farmland in North China

**Figure 5:**
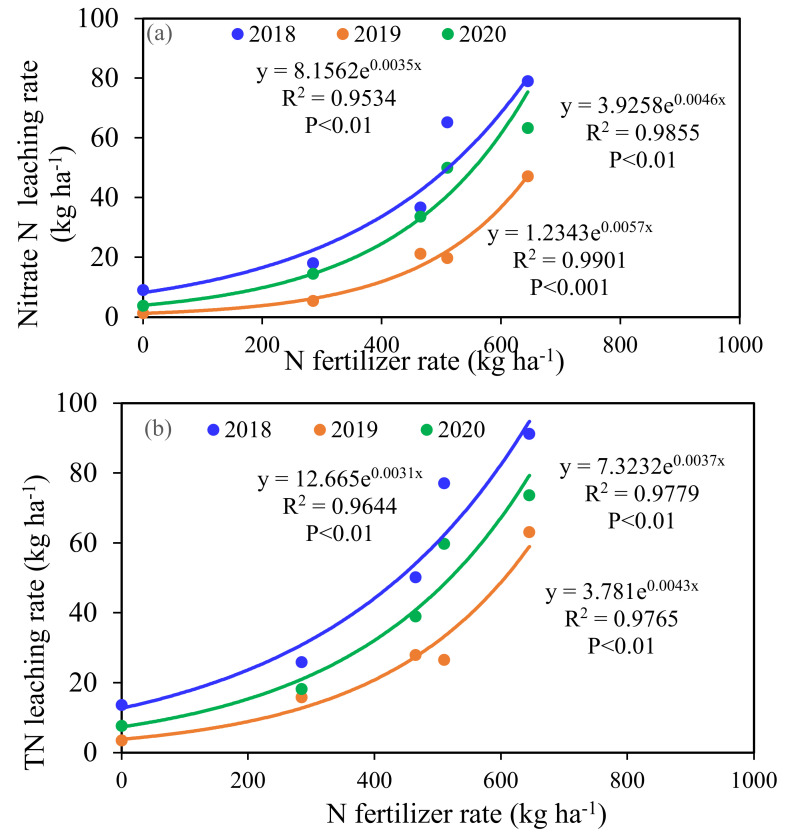
Correlations between the N fertilizer rate and N leaching over three rotations. (**a**) Correlation between the rate of N and nitrate N leaching; (**b**) correlation between the rate of N and TN leaching.

When N fertilizer was not applied, the concentration of nitrate N in the soil leaching solution at 90 cm was lower than the standard of underground drinking water established by the WHO. With the increase in N application rate, the concentration of N in the soil leachate increased substantially, and the concentrations of nitrate N in the soil leachate of LN, MN, MNO, and HN were 1.49-, 3.65-, 5.55-, and 7.57-fold those of the WHO standard for underground drinking water (Figure 2). A significant exponential relationship was observed between the rate of N fertilizer and N (nitrate N and TN) and that of leaching during the three-year period (Figure 5). In a wheat-maize rotation year, when the annual input of N < 300 kg N ha^−1^ is applied, the risk of N leaching is limited. However, when the N input is > 300 kg ha^−1^, N leaching increases exponentially. Although the N leaching amount of MN clearly increased compared with that of LN, the average nitrate N leaching amount of MN decreased by 51.7% compared with that of HN. LN had the lowest risk of N leaching in the four modes of N application. The relationship between N leaching and the rate of N application has been quantified through meta-analyses. Lu et al. [29] identified a significant linear relationship between N application and the leaching of nitrate N in wheat and maize cropland in China, although their studies were not meta-analyses. In contrast, Wang et al. [30] used a meta-analysis based on global nitrate leaching data to show that nitrate leached exponentially rather than responding linearly to increasing inputs of N. However, the leaching coefficient for specific crops was not determined. In this study, the nitrate N leaching coefficient was 4.4% when the annual N application rate was 285 kg ha^−1^ and 9.8% when the annual N application rate was 645 kg ha^−1^ (Figure 3).

**Figure 6:**
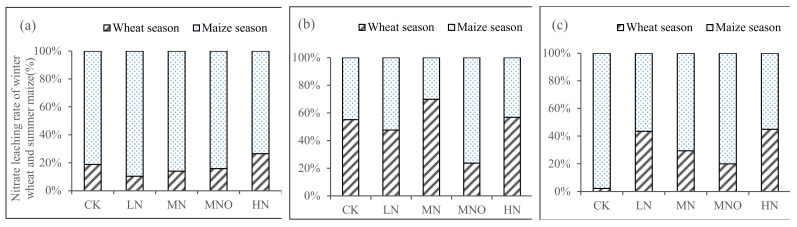
Nitrate leaching rate of winter wheat and summer maize in three rotation cycles. (**a**) RS1; (**b**) RS2; and (**c**) RS3. Note: CK, 0 N rate; LN, N input that was assumed to be less than the optimal application of N; MN, assumed to be the optimal N rate; MNO, additional organic fertilizer added to the rates of MN. HN, excess of N fertilizer.

Nitrate leaching is a complicated process that is affected by a complex set of interrelationships, including rainfall patterns, irrigation, fertilization management practices, and soil type [9,31,32]. Rainfall and irrigation are the main inputs of water in this study area. Nitrate N leaching was lower in the drier RS2, as indicated by the exponential relationships between N fertilizer and N leaching (Figure 3 and Figure 5). In contrast, higher rainfall promoted N leaching in wetter years (RS1 and RS3). The maize season received more rainfall than that of the wheat owing to the monsoon climate, thus increasing the leaching events and N leaching rate during this season. Furthermore, the loss of reactive N primarily occurred in the maize season during the rotation of winter wheat and summer maize in north China [26,33]. Li et al. [34] found that irrigation mainly affects water drainage and nitrate leaching during the winter wheat season. Our study showed that at RS1 and RS3, nitrate N leaching in the maize season accounted for a high proportion of the annual leaching, with average ratios of 83% and 72%, respectively. The average leaching amount of RS2 during the maize season accounts for 49.4% of the entire year (Figure 6). The use of high amounts of irrigation during the wheat season could also promote N leaching loss, and its contribution could be higher than that in the maize season.

After three years of research, we confirmed that nitrate N comprises the major part of N leaching in the cropland. In the present study, the average rate of nitrate N leaching to that of TN ranged from 50.6% to 82.4%. With the increase in N fertilizer use, the nitrate N leaching ratio to TN increases.

### 4.2. Effects of N Fertilizer Application on Soil Nitrate N Residue and N Utilization Rate

The soil nitrate N residue is closely correlated with the N fertilizer application. LN had a low concentration of nitrate N in the 2-m soil layer, and both the soil nitrate N residues in the soil layers that were 0–1 and 1–2 m deep had a concentration of less than 150 kg ha^−1^. MN, MNO, and HN increased the nitrate N residues in the 2-m soil layer during the two sampling periods, in which the values were 2.35-, 2.73-, and 3.02-fold of the LN, respectively (Figure 4). A significant exponential relationship between the N application rate and the average residual nitrate N was observed in 2 m soil (*y* = 121.8 e^0.0029x^; *R^2^* = 0.96). By summarizing the findings of research conducted in the Hebei and Shandong Provinces, the nitrate N residue in 1 m of soil was close to 300 kg ha^−1^ with a rate of N application >300 kg ha^−1^ in the wheat or maize season [29], which was lower than the results of this study. A higher accumulation of soil nitrate N under different treatments is mostly correlated with a relatively lower amount of rainfall in RS2. MNO had lower N leaching in RS2 and a higher one in RS3, suggesting that an increase in the application of organic fertilizer can delay the leaching of soil N in drought years. At RS2, MNO had a high concentration of nitrate N in the soil leachate, but the amount of nitrate N that leached out of the soil was low, primarily because the leaching amount of the soil leachate was reduced. The results of this study are consistent with the results of some studies, in which the application of organic fertilizer can improve the soil water holding capacity and reduce the leaching of nitrate N [35,36].

In the present study, HN had the lowest PFP. Although HN promoted N uptake in the plant, the N RE remained low. LN had the highest PFP and increased RE in both the wheat and maize seasons. MN also had increased RE and PFP. MN and LN reduced the leaching of N and improved the PFP and RE.

### 4.3. Optimization of the Recommended Amount of N Fertilizer and a Risk Index of N Leaching Losses in this Region

Many studies have been conducted in this region to reduce N leaching and N emissions losses by optimizing N application. However, the recommendations for N fertilizer can be distinguished from each other under respective field conditions. Manevski et al. [16] found that 300 kg ha^−1^ (100 for maize and 200 for wheat) was the optimal N application to achieve high yields and reduce nitrate leaching. A similar rate of N application of 190 kg ha^−1^ was also determined for winter wheat in the Huang-Huai-Hai Plain by Huang et al. [37], with a higher rate of 150 kg ha^−1^ for summer maize. Considering the yield and environmental effects, the optimal rate of N fertilizer should be approximately 185 kg N ha^−1^ for wheat cultivation, and this value will result in a yield of 7,000 kg ha^−1^ [38]. Zhang et al. [25] found that a range of N application of 150–240 kg ha^−1^ could maintain a high yield of maize and control nitrate N leaching < 18.4 kg ha^−1^ in north China by using a simulation model.

The recommended N use values of Chen et al. [33] were 220 and 256 kg N ha^−1^ for wheat and maize, respectively, which were based on experiments that covered the primary agroecological areas by considering integrated soil–crop systems. Moreover, the difference in recommended N fertilizer for the wheat season was relatively small, while the difference in recommended N fertilizer for the maize season was high. MN can be used to optimize the N application rate in this region, which can maintain annual nitrate N leaching of 30 kg ha^−1^, wheat and maize yield of 17.9 ton ha^−1^, and partial N fertilizer productivity of 38.4 kg ka^−1^. Furthermore, LN can be used as the lower limit for optimal N application in this region, which can maintain the annual nitrate leaching of 12.6 kg ka^−1^, total wheat and corn yield of 16.4 ton ha^−1^, and partial N fertilizer productivity of 57.4 kg ka^−1^ (Figure 4; Table 2 and Table 3).

## 5. Conclusions

An in situ leakage pond method was used to explore the characteristics of N leaching in a winter wheat-summer maize rotation in 2017–2020 in northern Henan Province. The soil nitrate N residue, yield of grain, and N utilization rate were also studied. We obtained the characteristics of N concentration in the soil leachate under different modes of N application. N leaching exponentially and significantly increased with N fertilizer use. The annual N application rate of 300 kg ha^−1^ was the critical point for the exponential growth of N leaching to transition from slow to rapid in wheat-corn rotation fields. The ratio of nitrate N leaching to total N leaching was obtained. N leaching and the N leaching loss coefficient in wetter years were significantly higher than those in drier years. A significant exponential relationship was observed between the 2 m soil nitrate N residue and the N application rate in one rotation cycle. We determined that the maize season was the primary period of N leaching in wheat-maize rotation fields, comprising 68.1% of the entire year on average. However, under the factors of high irrigation during the wheat season and climate, the amount of N leaching during the wheat season can also exceed 50% of the annual amount (RS2). The optimal N application rate of 285–465 kg ha^−1^ was determined for wheat-corn rotations in north China. On this basis, the risk of N leaching loss can be reduced while maintaining the high yield of crops.

## Figures and Tables

**Table 1:** Rate of fertilizer application of winter wheat and summer maize.

Treatment	Wheat (kg ha^−1^)			Maize (kg ha^−1^)		
	N	P_2_O_5_	K_2_O	N	P_2_O_5_	K_2_O
CK	0	90	90	0	67.5	67.5
LN	135	90	90	150	67.5	67.5
MN	225	90	90	240	67.5	67.5
MNO	270	135	135	240	67.5	67.5
HN	315	90	90	330	67.5	67.5

Note: CK, 0 N rate; LN, N input that was assumed to be less than the optimal application of N; MN, assumed to be the optimal N rate; MNO, additional organic fertilizer added to the rates of MN. HN, excess of N fertilizer.

**Table 2:** Grain yield of winter wheat and summer maize in three rotations.

Treatment	RS1 (kg ha^−1^)		RS2 (kg ha^−1^)		RS3 (kg ha^−1^)	
	Wheat	Maize	Wheat	Maize	Wheat	Maize
CK	7740.0 b	9114.0 b	2198.6 b	5797.2 c	4407.0 b	7293.8 b
LN	7990.0 b	9596.7 a	7209.7 a	7351.1 b	8006.9 a	8934.5 a
MN	8885.9 a	10,831.1 a	7477.1 a	9500.5 a	7853.4 a	9057.7 a
MNO	9094.3 a	10,470.0 a	7140.4 a	8309.4 a	7903.0 a	8622.0 a
HN	8694.0 a	9791.1 a	7952.5 a	9271.3 a	7843.5 a	9155.0 a

Note: Different letters in the same row indicate significant differences at the 5% level.

**Table 3:** N uptake and N utilization rate of winter wheat and summer maize.

Treatment	N uptake (kg ha^−1^)		RE (%)		PFP (kg kg^−1^)	
	Wheat	Maize	Wheat	Maize	Wheat	Maize
CK	87.7 c	120.8 c	–	–	–	–
LN	199.5 b	176.3 b	82.8 a	37.0 ab	57.3 a	57.5 a
MN	225.3 b	217.5 a	61.2 b	40.3 a	35.9 b	40.8 b
MNO	223.9 b	220.2 a	50.4 c	41.4 a	29.8 bc	38.1 b
HN	256.4 a	236.1 a	53.5 c	34.9 b	25.9 c	28.5 c

Note: N uptake and RE were calculated based on 2020 data, while PFP was calculated based on the average data for three years. Different letters in the same row indicate a significant difference at *p* < 0.05. RE, recovery efficiency; PFP, N fertilizer partial factor productivity.

## Data Availability

Not applicable.

## Supplementary Materials

The following supporting information can be downloaded at: https://www.mdpi.com/article/10.3390/plants11151963/s1, Figure S1. Monthly rainfall and irrigation management at the experimental site during the study period.
