# Supplementary material for: Optimal Fertilizer Application Reduced Nitrogen Leaching and Maintained High Yield in Wheat-Maize Cropping System in North China
Source: Plants (Basel). 2022 Jul 28;11(15):1963. doi: 10.3390/plants11151963 (PMC9370566; doi:10.3390/plants11151963)
Supplement: Supplementary file 1 [file plants-11-01963-s001.zip › plants-1812533-supplementary.pdf]

supplementary data

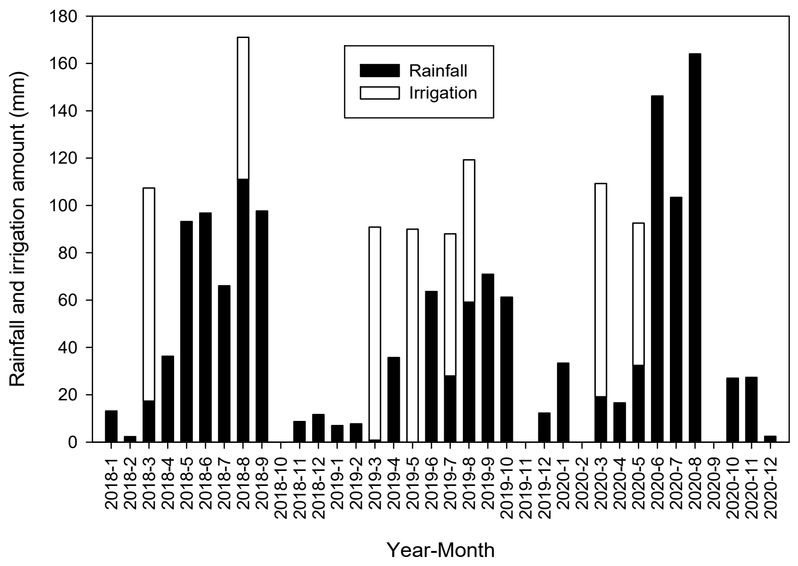

Figure S1. Monthly rainfall and irrigation management at the experimental site during the study period. In 2017/2018, irrigation was performed once for wheat and corn topdressing periods with irrigation amounts of 90 and 60 mm. The 2018/19 wheat season was irrigated once in March and May with 90 mm of water. Irrigation was performed once in July and August of the maize season with 60 mm of water. The 2019/20 wheat season was irrigated once in March and May, with irrigation amounts of 90 and 60 mm, respectively.
